# Viral Suppression and Resistance in a Cohort of Perinatally-HIV Infected (PHIV+) Pregnant Women 

**DOI:** 10.3390/ijerph13060568

**Published:** 2016-06-07

**Authors:** Maria Letícia Cruz, Edwiges Santos, Maria de Lourdes Benamor Teixeira, Monica Poletti, Carolina Sousa, Maria Isabel Gouvea, Karin Nielsen-Saines, Esaú João

**Affiliations:** 1 Hospital Federal dos Servidores do Estado, Rio de Janeiro 20221-161, Brazil; edwigesmotta@yahoo.com.br (E.S.); mlbenamor@hotmail.com (M.L.B.T.); monipoletti@hotmail.com (M.P.); cacau.nandes@gmail.com (C.S.); bebelsgouvea@uol.com.br (M.I.G.); esaujoao@gmail.com (E.J.); 2 School of Medicine, David Geffen University of California, 10833 Le Conte Avenue, Los Angeles, CA 90095, USA; KNielsen@mednet.ucla.edu

**Keywords:** HIV infection, pregnant women, vertical infection transmission, drug resistance, mutation

## Abstract

Our objective was to describe viral suppression and antiretroviral (ARV) resistance mutations in an ongoing cohort of perinatally-infected HIV+ (PHIV+) pregnant women. Descriptive analysis was performed using SPSS 18.0. From 2011 to 2014, we followed 22 PHIV+ pregnant women. Median age at prenatal entry was 19 years (Interquartile range (IQR) 17.6–21.0); 86% had an AIDS diagnosis; 81% had disclosed their HIV status to partner 11. The median age at HIV diagnosis was 8.3 y (IQR 4.0–13.6), the median age at sexual debut was 16 years (IQR 14–18). At the time of prenatal care initiation, four (18%) were on their first antiretroviral treatment (ART), eight (36%) in their second regimen and nine (41%) in their third regimen or beyond, and one had no data. Seventeen of 22 (77%) had HIV-viral load (VL) > 50 copies/mL at prenatal care entry, 16 had a genotyping exam performed. Seventeen of 22 PHIV+ had VL results near delivery: 7/17 (41%) had VL < 50 copies/mL. Among those who had genotyping at prenatal entry, 11/16 (69%) had mutations associated with ARV resistance. The most frequent major mutations were K103N, M184V, T215, M41L, D67N at reverse transcriptase gene and M46, I54V and V82A at protease gene. No vertical transmissions occurred. Management of pregnancy among PHIV+ is challenging. Individualized ART are needed to achieve viral suppression in a highly ART-exposed subpopulation.

## 1. Introduction

Individuals with perinatally-acquired human immunodeficiency virus type 1 (HIV) infection (PHIV+) are frequently diagnosed and started on antiretroviral therapy (ART) during the first months or years of life. The global expansion in the use of pediatric ART has resulted in a significant decrease in mortality rates among PHIV+, many of whom are now sexually active adolescents or young adults [1,2,3,4]. Very high viral loads during childhood and frequent family difficulties with sustaining high adherence to complex therapeutic regimens represent barriers to achieving and maintaining viral suppression in this population. An important consequence of this conjunction is the selection of resistant virus while successive changes in ART regimens occur during childhood [3,5,6,7,8]. When reaching adolescence, the need for a third line or salvage ART regimen is not rare among PHIV+. On the other hand, it has been observed that these adolescents have reproductive expectations similar to those of HIV-uninfected individuals [9,10], and limited therapeutic options for multi-drug resistant virus may affect the management of their treatment during pregnancy [11].

A resistance mutation is a mutation in a virus gene that allows the virus to become resistant to treatment with a particular antiviral drug. The term was first used in the management of HIV, the first virus in which genome sequencing was routinely used to look for drug resistance. Most antiretroviral drugs target HIV enzymes encoded by specific genes, reverse transcriptase, protease, or integrase. These enzymes are processed protein products of the HIV pol gene, a viral structural protein [12].

Our main objective was to describe viral suppression and ARV resistance mutations in an ongoing cohort of PHIV+ pregnant adolescents and women in Rio de Janeiro, Brazil.

## 2. Materials and Methods

We analyzed pregnancy data among confirmed PHIV+ women followed at a referral unit for prevention of mother-to-child transmission (PMTCT). Sociodemographic and clinical data, including treatment data, were collected routinely upon enrollment and during follow-up. Women with viral load (VL) > 1000 copies/mL at enrollment underwent HIV genotyping testing using the TRUGENE HIV-1 DNA sequencing platform. This platform detects HIV genomic mutations in the protease and reverse transcriptase regions of HIV, that are the most common mutations found among antiretroviral experienced individuals in our clinical settings [13]. The genotyping kit is a sequence-based assay target at codons 1–99 at the protease region and codons 40–247 at the reverse transcriptase region. Relevant ARV genotypic mutations were determined using the Stanford database. Descriptive analyses were performed using SPSS 18.0. This study was approved by the local Institutional Review Board (Approval Code 46937015.8.0000.5252).

## 3. Results

From February 2011 to August 2014, 630 HIV-infected pregnant women were enrolled in the PMTCT program. Among these, 22 (3.5%) were PHIV+. The median age at enrollment for the 22 PHIV+ was 18.5 years (Interquartile Range (IQR): 17.6–21.0), 71% were non-white, 86% had an AIDS-defining diagnosis according to CDC criteria, and 81% had disclosed their HIV status to the current sexual partner. The median number of years of formal education was nine years (IQR: 6.5–11).

The median age at HIV diagnosis was 8.3 years (IQR: 4.0–13.6), the median age at sexual debut was 16 years (IQR: 14–18), and 71% conceived while using antiretrovirals (ARV). At enrollment, 4/22 (18%) were on their first ART regimen, 8/22 (36%) on their second regimen, and 9/22 (41%) on at least their third ART regimen (1/22 had no ARV data available). Prenatal care was initiated at a median gestational age of 14 weeks (IQR: 9.0–20). At enrollment, 17/22 (77%) had a viral load (VL) > 50 copies/mL, and 17/22 (77%) had a VL assay result near delivery with 7/17 (41%) having a VL < 50 copies/mL at that time. Sixteen women had viral genotype testing at prenatal entry, and all received highly active antiretroviral therapy (HAART) during pregnancy. Table 1 shows the resistance profiles for these 16 PHIV+ subjects. Among those who had genotyping testing at enrollment, 11/16 (69%) had mutations associated with ARV resistance. The most frequent major mutations found among PHIV+ at enrollment were K103N (8/16, 50%) for NNRTIs; M184V (6/16, 38%), T215 (4/16, 25% ), M41L (3/16, 19%), and D67 (3/16, 19%) for NRTIs, and M46 (3/16, 19%), I54V (2/16, 13%) and V82A (2/16, 13%) for PIs.

All patients with VL > 50 copies/mL near delivery had a cesarean section. Among those with VL < 50 copies/mL, three had a vaginal delivery and four had a cesarean section for obstetric indications. There was one spontaneous abortion.

Infants’ mean gestational age was 38 weeks (IQR: 37–39), there were four preterm infants (18%). Mean birth weight was 2814 gr (IQR: 2.43–3.17) and mean birth length was 47 cm (IQR = 46–49). None of the infants were HIV-infected.

## 4. Discussion

A small proportion of pregnant women followed at our PMTCT unit acquired their HIV infection perinatally, and this analysis updates information available regarding this specific population. The main limitation of our study is the small sample, due to the rarity of this event, however this is a population of significant interest as more perinatally infected individuals have children of their own.

The median age of pregnant PHIV+ was similar to what was recently observed in the United States and Italy [11,14]. The median age of 8.3 years at HIV diagnosis was very close to 7.7 years reported for Italian pregnant PHIV+ [12], but slightly younger than age 10 years which we had observed in 2010 [4], pointing to a longer exposure time of ART use in the present population.

Among this group of PHIV+, we found a higher prevalence of diagnosis disclosure to sexual partners, as compared to PHIV+ in Africa and the UK/Ireland [10,15]. This is a very positive finding, which will likely positively impact the quality of life and outlook for these patients and newly established families.

At enrollment, only 5/22 (23%) of PHIV+ had a VL below 50 copies/mL, but close to delivery this percentage increased to 41%. Our results are comparable to those from a US cohort that found 60% of pregnant PHIV+ with a VL below 400 copies/mL [11] and worse than a cohort from the United Kingdom where only 33% of PHIV+ pregnant had a viral load above 50 copies/mL near delivery [14].

Most of our patients have been exposed to at least two ART regimens and the observed suboptimal viral suppression among PHIV+ at enrollment is probably associated with non-adherence to ART and the presence of viral resistance. In a systematic review that included studies published from 1990 to 2011 with 1980 PHIV+ on ART from 26 countries, Sánchez *et al.* [16] found drug resistance mutations in 78% of PHIV+, higher than the 69% noted in our study. These observations among perinatally-infected populations differ significantly from data in the general population of HIV-infected women. Our own group has reported ART resistance mutations in 15.6% of 231 HIV-infected women during pregnancy [13]. In our cohort, 8/16 (50%) and 3/16 (19%) had NNRTI and PI resistance mutations, respectively. Data from 211 adolescents in the United Kingdom exposed to ART showed 65% had mutations for NNRTIs, mainly K103N and Y181C, and 26% with mutations for PIs [3]. K103N mutation is in the reverse transcriptase gene region of HIV-1. It causes the hydrophobic pocket in which the NNRTI binds to inhibit enzyme activity in reverse transcriptase to close by means of a hydrogen bond. K103N is a nonpolymorphic mutation selected in patients receiving NVP and EFV. It reduces NVP and EFV susceptibility by about 50 and 20-fold, respectively [17]. Similarly, M184V confers high-level resistance to lamivudine, and is a discriminatory mutation. It occurs at or near the binding site of the reverse transcriptase gene, a common feature of mutations that confer resistance to NRTIs [12]. T215 confers resistance to zidovudine, and is also in the reverse transcriptase gene. It is one of the Thymidine Analog Mutations (TAMs), similarly to M41L, which is also a mutation conferring high level resistance to zidovudine, stavudine and some resistance to didanosine, abacavir and tenofovir. D67 is also an NRTI mutation in the RT gene, which confers resistance to zidovudine and stavudine. M46 is a protease inhibitor mutation associated with resistance to lopinavir/ritonavir and atazanavir/ritonavir. It is an HIV protease gene mutation [18]. 154 V is a mutation associated with protease inhibitor resistance, particularly nelfinavir [19]. Like the others, is located in the HIV pol gene. V82A is a mutation also associated with protease inhibitor resistance in the HIV protease region [20]. 

The gestational age of infants born to PHIV+ are similar to that described in cohorts in Italy and New York [12,21] where the proportions of preterm infants were 38% and 29%, higher than what we observed. Mean birth weight among our patients was higher than in these groups probably due to the smaller proportion of preterm infants.

## 5. Conclusions 

Despite advances in HIV treatment, ART management during the pregnancies of PHIV+ is especially challenging. New drugs, effective counseling, strategies and interventions targeting patient retention and individualized ART regimens are needed and may be crucial to achieve viral suppression in a highly ART-exposed subpopulation with expected adherence difficulties.

## Figures and Tables

**Table 1:** Genotypic resistance mutations identified and antiretroviral resistance profile in pregnant women with perinatally-acquired HIV infection.

Patient Number	Subtype	Protease Inhibitors (PI)		Reverse Transcriptase Inhibitor Mutations		Resistance Profile		
		Major Mutations	Minor Mutations	NRTI	NNRTI	Low	Intermediate	High
**1**	NR	0	L10V	0	0			
**2**	B	0	L10V	0	0			
**3**	B	0	0	0	0			
**4**	B	0	K20T A71T	M41L M184V T215D	K101P K103NS Y188HL	TDF NFV	ABC ZVD D4T DDI	3TC FTC EFV ETR NVP RVP
**5**	F1	0	L10I	M184V T215Y	V90I K103N E138A	DDI RPV	ABC ZVD D4T	3TC FTC EFV NVP
**6**	B	0	0	D67N T69D M184V K219Q	A98G Y181C H221Y	ABC ZVD D4T	DDI EFV ETR RPV	3TC FTC NVP
**7**	B	V32I M46I I47A	L10I A71V	M41L D67N V75M M184V L210W T215Y K219N	K103N		ATZ/r DRV/r IDV/r TPV/r	ABC ZVD D4T DDI 3TC FTC TDF EFV NVP FSP/r NFV LPV/r
**8**	F1	0	L10V	0	K103N			EFV NVP
**9**	B	0	0	0	0			
**10**	B	M46L I54IV V82AV L90LM	L10F L33F G73AG	M41L T215S K219R	K103N	3TC FTC DRV/r	ABC DDI TDF TPV/r	ZVD D4T EFV NVP ATZ/r FSP/r IDV/r LPV/r NFV SQV/r
**11**	F1	0	0	0	K103N V106I			EFV NVP
**12**	B	M46I I54V L76V V82A	L10V A71V	D67N T69N K70R M184V K219Q	0	TDF DRV/r TPV/r	ABC D4T DDI ATZ/r SQV/r	ZVD 3TC FTC FSP/r IDV/r LPV/r NFV
**13**	B/F	0	A71V	0	0			
**14**	F1	0	0	D67N K70R M184V K219Q	K103N P225H	TDF	ABC AZT D4T DDI	3TC FTC EFV NVP
**15**	B	0	0	M184V	K103N	DDI ABC		3TC FTC EFV NVP
**16**	B	0	0	M184V	V179D Y188L	ABC ETR		3TC FTC EFV NVP RPV

## Abbreviations

The following abbreviations are used in this manuscript:

PHIV+	perinatally-HIV infected
ARV	Antiretroviral
ART	Antiretroviral therapy
AIDS	Acquired Immunodeficiency Syndrome
HIV	Human Immunodeficiency Virus
VL	Viral load
CDC	Centers for Disease Control and Prevention
IQR	Interquartile range
NNRTI	Non-nucleoside reverse transcriptase inhibitor
PI	Protease inhibitor
TDF	Tenofovir
NFV	Nelfinavir
DDI	Didanosine
RPV	Rilpivirine
ABC	Abacavir
ZVD	Zidovudine
D4T	Estavudine
3TC	Lamivudine
FTC	Emtricitabine
DRV	Darunavir
TPV	Tripanavir
ETV	Etravirine
EFZ	Efavirenz
ATV	Atazanavir
IDV	Indinavir
SQV	Saquinavir
NVP	Nevirapine
FSP	Fosamprenavir
LPV	Lopinavir
